# Paper-based acetylcholinesterase inhibition assay combining a wet system for organophosphate and carbamate pesticides detection 

**DOI:** 10.17179/excli2014-684

**Published:** 2015-02-26

**Authors:** Amara Apilux, Chartchalerm Isarankura-Na-Ayudhya, Tanawut Tantimongcolwat, Virapong Prachayasittikul

**Affiliations:** 1 Faculty of Medical Technology, Mahidol University, Bangkok 10700, Thailand, Center for Innovation Development and Technology Transfer; 2 Faculty of Medical Technology, Mahidol University, Bangkok 10700, Thailand, Department of Clinical Microbiology and Applied Technology

**Keywords:** paper-based sensor, wet system, pesticides, acetylcholine inhibition

## Abstract

A dramatic increase in pesticide usage in agriculture highlights the need for on-site monitoring for public health and safety. Here, a paper-based sensor combined with a wet system was developed for the simple and rapid screening of organophosphate (OP) and carbamate (CM) pesticides based on the inhibition of acetylcholinesterase (AChE). The paper-based sensor was designed as a foldable device consisting of a cover and detection sheets pre-prepared with indoxyl acetate and AChE, respectively. The paper-based sensor requires only the incubation of a sample on the test zone for 10 minutes, followed by closing of the foldable sheet to initiate the enzymatic reaction. Importantly, the buffer loading hole was additionally designed on the cover sheet to facilitate the interaction of the coated substrate and the immobilized enzyme. This subsequently facilitates the mixing of indoxyl acetate with AChE, resulting in the improved analytical performance of the sensor. The absence or decrease in blue color produced by the AChE hydrolysis of indoxyl acetate can be observed in the presence of OPs and CMs. Under optimized conditions and using image analysis, the limit of detection (LOD) of carbofuran, dichlorvos, carbaryl, paraoxon, and pirimicarb are 0.003, 0.3, 0.5, 0.6, and 0.6 ppm, respectively. The assay could be applied to determine OP and CM residues in spiked food samples. Visual interpretation of the color signal was clearly observed at the concentration of 5 mg/kg. Furthermore, a self-contained sample pre-concentration approach greatly enhanced the detection sensitivity. The paper-based device developed here is low-cost, requires minimal reagents and is easy to handle. As such, it would be practically useful for pesticide screening by non-professional end-users.

## Introduction

Over the past decades, the use of pesticides has considerably increased worldwide, including in Thailand (Barr and Needham, 2002[3]; Panuwet et al., 2012[22]). Among the pesticides, organophosphates (OPs) and carbamates (CMs) have been extensively used in agriculture to protect crops against insect invasion. However, OPs and CMs are considered one type of neurotoxic compound. Their toxicity is based on the inhibition of acetylcholinesterase (AChE) in the central nervous system, resulting in the accumulation of a neurotransmitter, namely acetylcholine (Chapalamadugu and Chaudry, 1992[4]; Du et al., 2008[6]). This, in turn, causes neurotoxic symptoms including headache, increased salivation, convulsion, breathing suppression and even death. The inappropriate use of pesticides is a leading cause of pesticide contamination in food and environment and impacts human well-being. Thailand is an agricultural country where the utilization of OPs and CMs accounts for the major usage of pesticides. OP and CM residues exceeding the maximum residue levels (MRLs) have been found and have drawn extensive attention (Duangchinda et al., 2014[7]; Sapbamrer and Hongsibsong, 2014[24]). From our data, 12 types of pesticides in the OP and CM groups were detected in 85.5 % of the Chinese kale samples from the local consumer market. To ensure safe agricultural production and prevent toxicity, a simple, economic, and rapid analysis method is important as an early warning tool for the consumers. 

The conventional methods for the determination of pesticide residues involve gas chromatography (GC) or high performance liquid chromatography (HPLC) combined with mass spectroscopy (Fenik et al., 2011[9]; Sharma et al., 2010[25]; Zhao et al., 2011[28]). Although such methods provide high sensitivity and accuracy, they are sophisticated, time-consuming, and costly and require sample preparation procedures and highly skilled operators. In recent decades, numerous electrochemical (Andreescu and Marty, 2006[1]; Liu et al., 2013[16]; Mulchandani et al., 2001[18]; Prieto-Simón et al., 2006[23]; Vakurov et al., 2004[27]) and optical (Jin et al., 2004[14]; Liu et al., 2013[16]; Obare et al., 2010[21]; Simonian et al., 2005[26]) biosensors have been developed as alternative methods for pesticide detection. Nevertheless, these biosensors still require instrumentation and skilled operators, which is not suitable for practical application in some circumstances (e.g., home and field applications). Additionally, an immunochromatographic assay based on the binding of an antibody to a specific pesticide has also been developed (Liu et al., 2013[16]; Mallat et al., 2001[17]). However, the system is limited to certain types of pesticides and is unable to screen unknown pesticides. The test is also costly because of the expensive antibody used. Regarding the limitations of the existing methods, a simple on-site assay is necessary to facilitate the screening of pesticide contamination.

Paper-based devices are a new technology of total analysis systems equipped on paper or paper-like materials (Apilux et al., 2012[2]; Chen et al., 2012[5]; Dungchai et al., 2010[8]; Hu et al., 2014[13]). The devices are good potential alternative tools for on-site detection because they are easy to use, inexpensive, portable and rapidly implemented. Currently, several works related to AChE inhibition assay on paper have been carried out for pesticide analysis via the colorimetric quantification of the product of AChE-catalyzed hydrolysis. The change in color is dependent on the concentration of the pesticides that inhibit AChE activity. The assays were performed using different chromogenic substrates, including 5,5'-dithiobis-(2-nitrobenzoic acid) (DTNB) (Nagatani et al., 2007[19]), indophenyl acetate (Hossain et al., 2009[12]; No et al., 2007[20]), and indoxyl acetate (Guo et al., 2013[10]; Han et al., 2012[11]) with different formats and paper types. However, these methods require either a multi-manual operation with bidirectional lateral flow assays (Hossain et al., 2009[12]) or some degree of sample and reagent operation to complete the assay (Guo et al., 2013[10]; Nagatani et al., 2007[19]; No et al., 2007[20]). Thus, a novel design of paper-based device that simplify a multistep reaction is still required to achieve practical detection efficiencies of OP and CM residues. Although paper-based devices provide several advantages (the reagents for the assay can be pre-prepared on the paper and will readily interact with the sample), they provide relatively low detection sensitivities because of the limited mixing of the reagents on the paper. 

Here, a paper-based device with a wet system was developed to simplify the process of pesticide detection based on the AChE inhibition assay for practical usage. The sensor consists of an AChE-immobilized paper as the test zone and a cholinesterase substrate indoxyl acetate-coated paper attached on opposite sides of a foldable plastic sheet; a waterproof film was placed at the interface of the two paper sheets. Additionally, the wet system was newly created to enhance the sensitivity by providing a hole on the verso of the substrate sheet for the addition of a buffer after the enzymatic reaction. Furthermore, the paper-based sensor enables the pre-concentration of the sample at the test zone, which can improve the sensitivity of the detection of pesticides at very low concentrations. 

## Materials and Methods

### Conceptual design of paper-based pesticide sensor 

**Figure 1:**
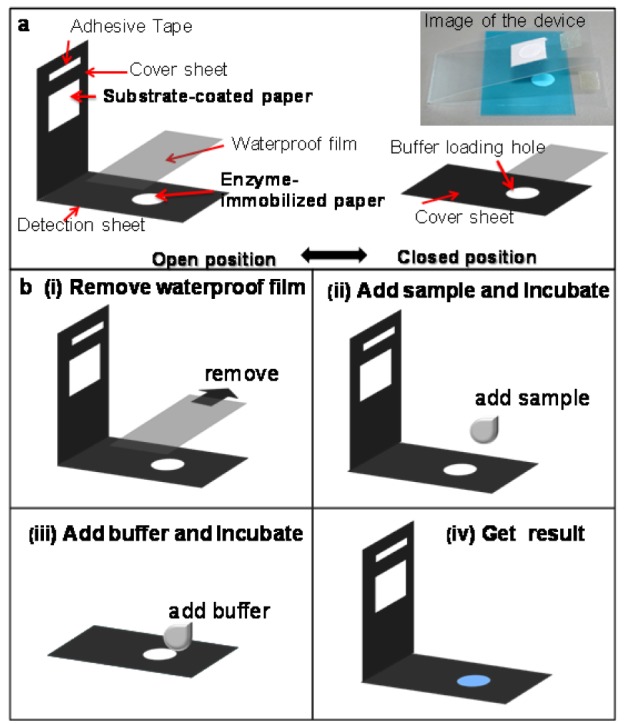
(a) Illustration of the paper-based AChE inhibition devices. (b) Schematic diagram of the pesticide detection process

The paper-based sensor based on the AChE inhibition assay combining a wet system is shown in Figure 1a(Fig. 1). The device was designed as a foldable sheet consisting of two main parts: 

1) an enzyme-immobilized paper attached on the detection sheet and 

2) a substrate-coated paper attached on the cover sheet. 

The sheets were separated by a waterproof film to prevent the undesired interaction of the dried reagents that were pre-spotted on each side of the sensor. The procedure of the paper-based AChE inhibition assay for pesticide detection is shown in Figure 1b(Fig. 1) and is briefly summarized in the following steps: 

(i) open the cover and remove the waterproof film; 

(ii) add the sample solution and incubate for 10 min at room temperature; 

(iii) close the cover and add the buffer solution twice at 5-min intervals; and 

(iv) open the cover to visualize color intensity after 10 min of incubation.

### Chemicals and materials

Acetylcholesterase (EC 3.1.1.7, from *Electrophorus electricus*), indoxyl acetate, sucrose, lauryl sulfate sodium and casein were purchased from Sigma-Aldrich. The pesticide standards of OPs (dichlorvos and paraoxon-methyl) and CMs (carbaryl, carbofuran, and pirimicarb) were obtained from Dr. Ehrenstorfer GmbH (Augsburg, Germany). Methanol, disodium hydrogen orthophosphate and sodium dihydrogen phosphate were purchased from BDH Prolabo (Lutterworth, UK). Analytical-grade reagents and 18 MΩ-cm water were used throughout this experiment. Filter paper No.1 (Whatman, UK) was used to make the reagent support materials that were attached on a polypropylene plastic sheet (purchased from a local stationery store).

### Preparation of reagents and paper-based AChE inhibition assay for pesticides detection

AChE was prepared at 800 U/mL in a 20 mM phosphate buffered saline solution (PBS, pH 7.4: 2.88 g Na_2_HPO_4_, 0.4 g KCl, 0.48 g KH_2_PO_4_, and 16 g NaCl in 800 mL dissolved in distilled water). The blocking solution was a 50 mM boric acid buffer (pH 8.3) with 0.5 % (w/v) casein, and the washing solution was a 50 mM phosphate buffer (pH 7.5) with 0.01 % (w/v) SDS. The 5 mg/mL of indoxyl substrate was prepared in methanol. The desired concentrations of the pesticide test solutions were prepared by diluting an appropriate volume of each stock pesticide (1,000 ppm in methanol) with 20 mM PBS buffer. 

The enzyme-immobilized paper was prepared by punching the filter paper in a circular shape with a 6 mm diameter. For immobilization, AChE (5 μL of 800 U/mL) was dropped onto the prepared paper and allowed to dry for 1 hour. The paper was blocked against non-specific binding by immersion in the blocking solution for 20 min at room temperature. Then, the blocked paper was washed by dipping in the washing solution for 30 min at room temperature, dried for 2 hours at room temperature, and kept at –20 °C until use. The substrate-coated paper was prepared by cutting the filter paper into 10 x 10 mm squares. Then, 20 μL of 5 mg/mL indoxyl acetate in methanol was spotted onto the prepared paper, dried at room temperature and kept in a sealed zipper-lock at –20 °C until use. The enzyme-immobilized paper and the substrate-coated paper were individually attached onto a plastic sheet at the opposite sites of a foldable platform by using double-sided adhesive tape. For the cover sheet, the substrate-coated paper was attached in a way that allowed for the diffusion of the buffer solution through the sensing zone. 

### Measurement of pesticides in food sample

The performance of the paper-based AChE assay for pesticide detection was verified against lettuce and brown rice samples. The different concentrations of pesticides were added onto pesticide-free lettuce leaves or brown rice grains in proportions of 1 mL/g. After equilibration for 24 hours, 1 g of lettuce leaves or brown rice grains was chopped or ground into fine pieces and then immersed into the 20 mM PBS buffer for 10 min. The supernatant was subjected to pesticide analysis by the paper-based device. The concentrations of pesticide in the samples were confirmed by gas chromatography and tandem mass spectrometry (GC-MS/MS) (Bruker SCION Triple Quadrupole Detector, Germany). To enhance the detection performance, the pesticide-spiked lettuce leaves and brown rice grains were extracted using the QuEChERS (Quick, Easy, Cheap, Effective, Rugged, and Safe) method (Lehotay et al., 2010[15]).

### Data processing and interpretation

The color signal generated by the reaction of AChE with the indoxyl acetate substrate can be determined by naked eyes for a qualitative analysis. Additionally, the image was captured using a digital camera (IXY200F, Canon, Japan) and transferred to the ImageJ 1.45s software (National Institutes of Health, USA) for a semi-quantitative analysis. The color signal at the test zone image was analyzed by measuring the mean intensity in the RGB channel. The mean intensity value of each test zone was obtained by subtracting the intensity from that of the background (the area above the test zone). Next, the background-subtracted intensity values were used to obtain a calibration curve.

## Results and Discussion

### Optimization of the experimental conditions

**Figure 2:**
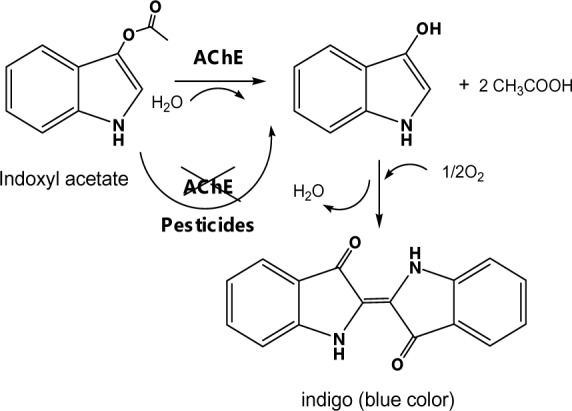
The hydrolysis mechanism of indoxyl acetate catalyzed by AChE

The paper-based pesticide sensor for the screening of OPs and CMs based on an AChE inhibition assay was newly developed, as shown in Figure 1(Fig. 1). The filter paper was chosen to serve as a supportive material for the immobilization of AChE, and an indoxyl substrate because of its inexpensive cost and efficient handling of pre-treated reagents. The colorimetric measurement involves the catalytic hydrolysis of indoxyl acetate by AChE, which afterward produces a blue color according to the reaction shown in Figure 2(Fig. 2). The pesticide detection is based on the inhibition of AChE activity by OP and CM pesticides, which reduces the color intensity of the hydrolyzed indoxyl acetate and can be observed by the naked eye and with an image analysis software. 

**Figure 3:**
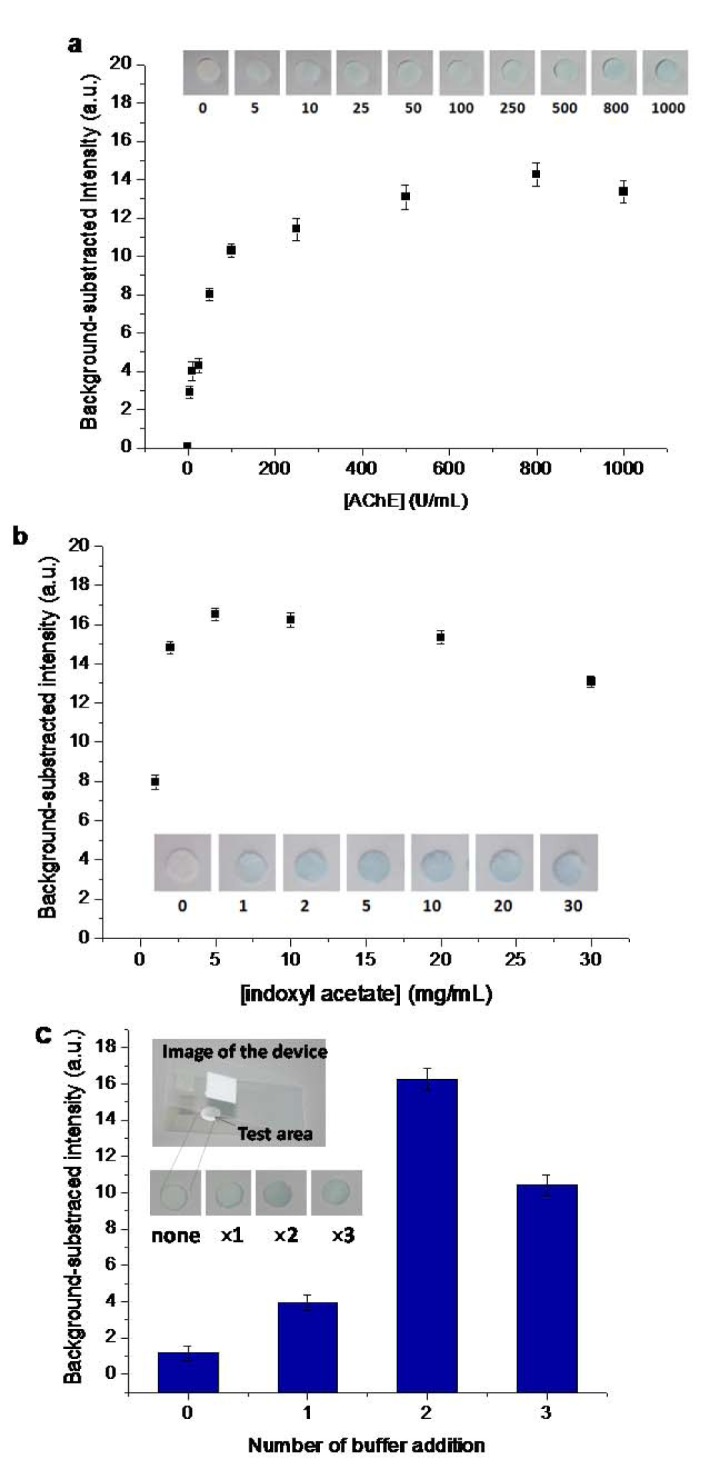
Optimization of the hydrolysis of indoxyl acetate by AChE. (a) Effect of AChE concentrations with 20 L of 30 mg/mL indoxyl acetate. (b) Effect of indoxyl acetate concentrations with 800 U/mL AChE. (c) Effect of wet system on color intensity with multiple applications of 10 L of the buffer solution. The hydrolysis is represented as background-subtracted intensity determined by digital-image analysis using Image J. Insets represent results at sensing area. Data are the means ( the standard deviation) of 3 independent measurements.

The sensitivity of the AChE inhibition assay on paper-based sensors is affected by various factors including enzyme and substrate concentrations, wetness, and incubation time. Therefore, the optimal conditions for the analysis of pesticides were determined. The concentration of AChE was varied between 5 to 1,000 U/mL, and 5 μL of the enzyme solution was immobilized on the filter paper. Subsequently, 10 μL of 20 mM PBS buffer was added and allowed to dry for 5 min at room temperature. The color intensity was developed by adding 30 mg/ mL of indoxyl acetate. The result is shown in Figure 3a(Fig. 3). The color intensity increased with the increase in AChE concentration and was almost constant when the AChE concentration was above 800 U/mL. Therefore, an AChE concentration of 800 U/mL was selected for the subsequent experiments. The optimal concentration of indoxyl acetate substrate was investigated in the range of 1 to 30 mg/mL in 20 μL (Figure 3b(Fig. 3)). The color intensity increased with increasing indoxyl acetate concentration. A slight decrease in color intensity was observed at indoxyl acetate concentrations higher than 5 mg/mL, which was therefore selected as the optimal indoxyl acetate concentration. 

Subsequently, under the optimized conditions mentioned above, the AChE-coated paper and substrate-coated paper were constructed on a foldable plastic sheet as shown in Figure 1(Fig. 1). However, there is a limitation to this paper-based assay: the reaction is inefficient because of the ineffective interaction among the pre-deposited reagents on the paper under dry conditions. Therefore, a novel and simple wet system was developed and integrated into the paper-based sensor. The assay was performed by repeated applications of the buffer solution onto the sensing area through a hole in the cover sheet after the incubation of the sample. The results (Figure 3c(Fig. 3)) indicated that the enhanced color intensity was obtained by increasing the number of applications of the buffer solution and waiting 5 min between the drops. A double loading of the buffer was found appropriate for color development. This is due to the wet assay allowing for the efficient mixing of the pre-deposited reagents on the paper-based substrate. Thus, this approach was subsequently applied for further testing.

### Effect of inhibition time

**Figure 4:**
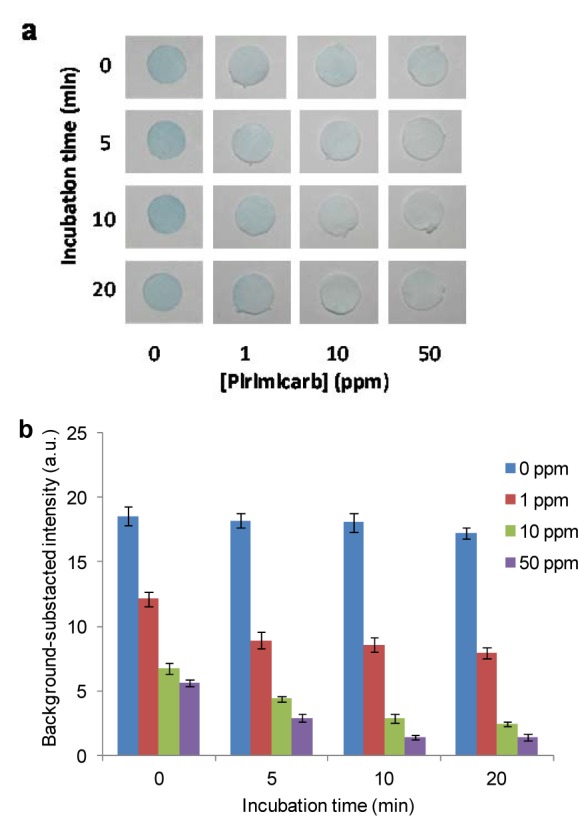
Effect of incubation time on pesticides detection by the developed device: (a) Measurement of pirimicarb at 0, 1, 10, 20 ppm with different incubation times. (b) Plot of background-subtracted intensity determined by digital-image analysis versus the concentrations of the added pirimicarb solutions; represent to (a).

Pirimicarb was selected as a representative pesticide to investigate the effect of incubation time on the efficiency of the AChE inhibition assay. The process was performed by adding 10 mL of various concentrations (1, 10, and 50 ppm) of pirimicarb to the test zone, followed by incubating for 0, 5, 10 or 20 min. Subsequently, 10 mL of 20 mM PBS buffer was added twice to the test zone via the buffer adding hole. The results showed that the color intensity of the detection zone decreased with the increase in the concentration of pirimicarb. The color change was clearly observed when the incubation time was increased up to 10 min, after which the response remained steady (Figure 4(Fig. 4)). Therefore, the sample solution was incubated with the enzyme-immobilized paper for 10 min at room temperature before being subjected to the hydrolysis reaction of the substrate. 

### Performance of the paper-based AChE inhibition assay for pesticides detection

**Figure 5:**
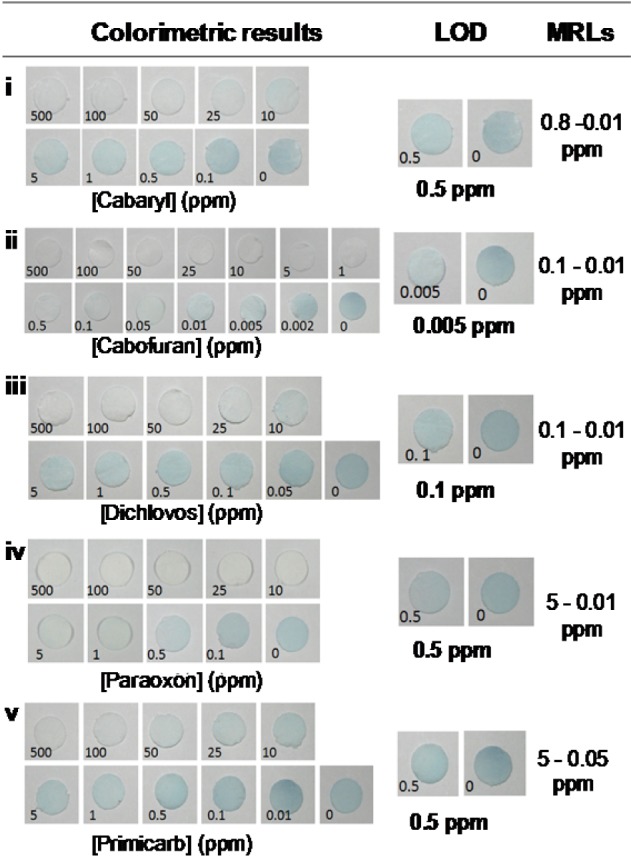
Determination of limit of detection (LOD) of the paper-based device for pesticide screening. A representative visual image of AChE inhibition on the sensing areas with various concentrations of (i) carbaryl, (ii) carbofuran, (iii) dichlorvos, (iv) paraoxon and (v) pirimicarb. Image shown in representative of those seen in three independent repeats.

The performance of the paper-based AChE inhibition device was examined against concentrations of OPs (dichlovos and paraoxon) and CMs (carbaryl, carbofuran, and pirimicarb). The intensity of the blue color at the test zone dramatically decreased with the increase in concentration of insecticides, which could easily be observed by the naked eye after 10 min of assay (Figure 5(Fig. 5)). The saturated inhibition concentration of carbaryl, carbofuran, dichlovos, paraoxon, and pirimicarb was found to be 50, 1, 50, 10 and 100 ppm, respectively. The limit of detection (LOD) by visual detection was estimated to be 0.5 ppm of carbaryl, 0.005 ppm of carbofuran, 0.1 ppm of dichlovos, 0.5 ppm of paraoxon and 0.5 ppm of pirimicarb. The variation in sensitivity is due to the difference in the biomolecular rate constant of AChE inhibition in the pesticides. The limit of detections provided by our developed device were slightly superior than that of previous reports, which were sensitive enough for the screening of pesticide residues in contaminated agricultural products to meet the maximum residual concentration (MRL) of pesticides declared by the European Union (EU) (http://ec.europa.eu/sanco_pesticides/public/?event=homepage) and Japan (JA) (http://www.m5.ws001.squarestart.ne.jp/foundation/search.html). The recommended MRLs vary among the pesticides within each food group because the MRLs are set based on the permissible use of a pesticide on a crop under the Good Agricultural Practice (GAP), the expected residues and the toxicological reference values of each pesticide (e.g., chronic toxicity and acute toxicity).

**Figure 6:**
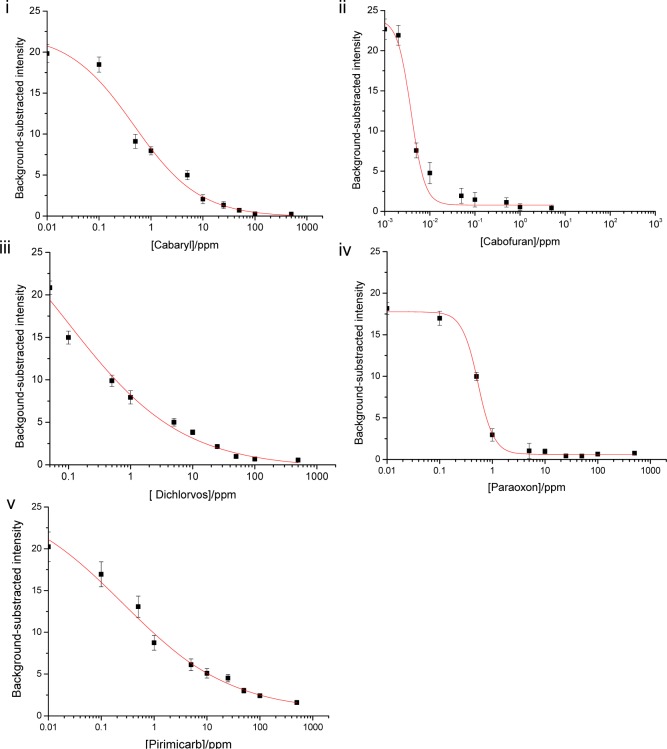
Plots of mean color intensity values determined by digital image analysis using Image J and the concentrations of the added pesticides solutions (i) carbaryl, (ii) cabofuran, (iii) dichlorvos, (iv) paraoxon and (v) pirimicarb, from three independent repeats. The curves were fitted by the Hill equation. Data are the means (± the standard deviation) of 3 independent measurements.

To obtain more reliable measurements, the semi-quantitative determination of color intensity was performed using the ImageJ software analysis. Figure 6(Fig. 6) shows the calibration curves obtained by plotting the background-subtracted color intensity versus the standard concentrations of each pesticide. The limit of detection is given by IC_50_, which was the concentration of pesticide that inhibited 50 % of the AChE activity. The LODs were found to be 0.003 ppm of carbofuran, 0.3 ppm of dichlorvos, 0.5 ppm of carbaryl, 0.6 ppm of paraoxon and pirimicarb. The results indicated that the visual detection provided a good detection performance, comparable to the semi-quantitative analysis. 

### Detection of OP and CM residues in food samples

**Figure 7:**
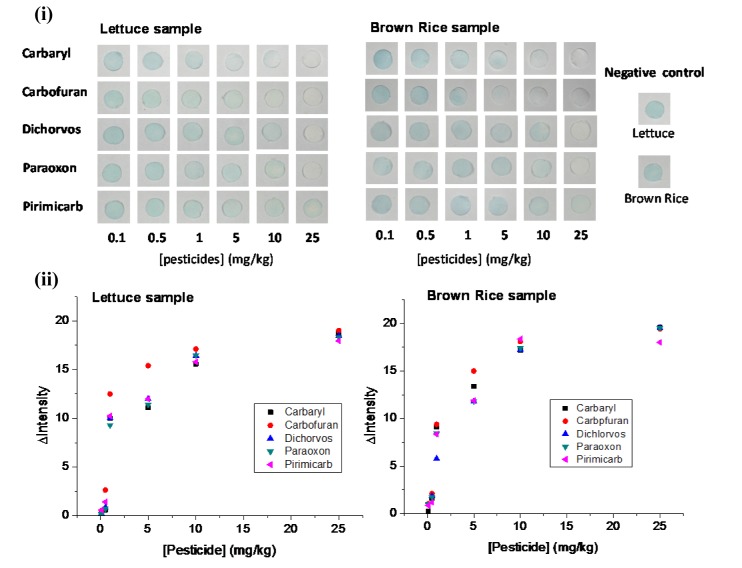
Detection of pesticide residues at different concentrations in lettuce and brown rice. The results are presented as (i) photographs and (ii) plots of the mean intensity of the sensing area on the paper-based sensor versus pesticide concentration.

To investigate the performance of the paper-based sensor in determining pesticide residues in real samples, the approach was applied to the screening of OPs and CMs in lettuce and brown rice. The confirmed pesticide-negative lettuce and brown rice were spiked with various concentrations of each pesticide (0, 0.1, 0.5, 1, 5, 10, and 25 mg/kg) and exposed to the paper-based pesticide sensor under the aforementioned optimal assay conditions. The color intensity of the paper-based sensor in response to the pesticides was quantitated by the ImageJ software and expressed in terms of the color intensity difference (ΔI) between the blank sample (I_blank_) and the pesticide spiked sample (I_spiked_). The plots of ΔI (I_blank _– I_spiked_) versus pesticide concentration are shown in Figure 7(Fig. 7). The ΔI increased with pesticide concentration and the color intensity change could be easily distinguished by the naked eye when concentrations of pesticides were above 5 mg/kg. The semi-quantitative analysis by image processing provides a LOD of 1 mg/kg. The diminution of sensitivity toward OP and CM residues in lettuce and brown rice samples is due to either the poor recovery of the pesticides or the interference effect of sample matrix. 

The analytical recoveries of the spiked pirimicarb at 0.5 and 5 mg/kg in rice and lettuce samples were evaluated using Hill’s equation. The sample solution was subjected to the extracted pesticide residue by using 2 different protocols: (1) immersion in 20 mM PBS buffer at pH 7.4 and (2) the QuEChERS extraction method. In the case of immersion with PBS buffer, the optimum percentage of methanol added in the buffer was investigated to improve the recovery. The effect of methanol on the enzyme activity was studied, and 20 mM PBS at pH 7.4 in combination with 20 % methanol was selected for subsequent study (data not shown). The results of the analytical recoveries are shown in Table 1(Tab. 1). 

The QuEChERS extraction method with a paper-based sensor gave excellent results that were in a good agreement with QuEChERS and GC/MS-MS analyses. The analytical recoveries ranged between 88 and 110 %. 20 mM PBS at pH 7.4 in combination with 20 % methanol provided analytical recoveries ranging between 62 and 69 % while 20 mM PBS at pH 7.4 gave 31–38 % recovery. Moreover, this assay exhibited precisions (% RSD) in the range of 5.8 to 14.8 % and 6.4 to 15.7 % for lettuce and rice, respectively. 

These results indicated that the matrix could affect the accuracy of the method. Thus, this approach is able to perform pesticide screening. However, when the MRL values in food are lower than 1 mg/mL, the QuECHERs procedure is required. 

### Enhancement of pesticide detection performance by the self-contained pre-concentration method

**Figure 8:**
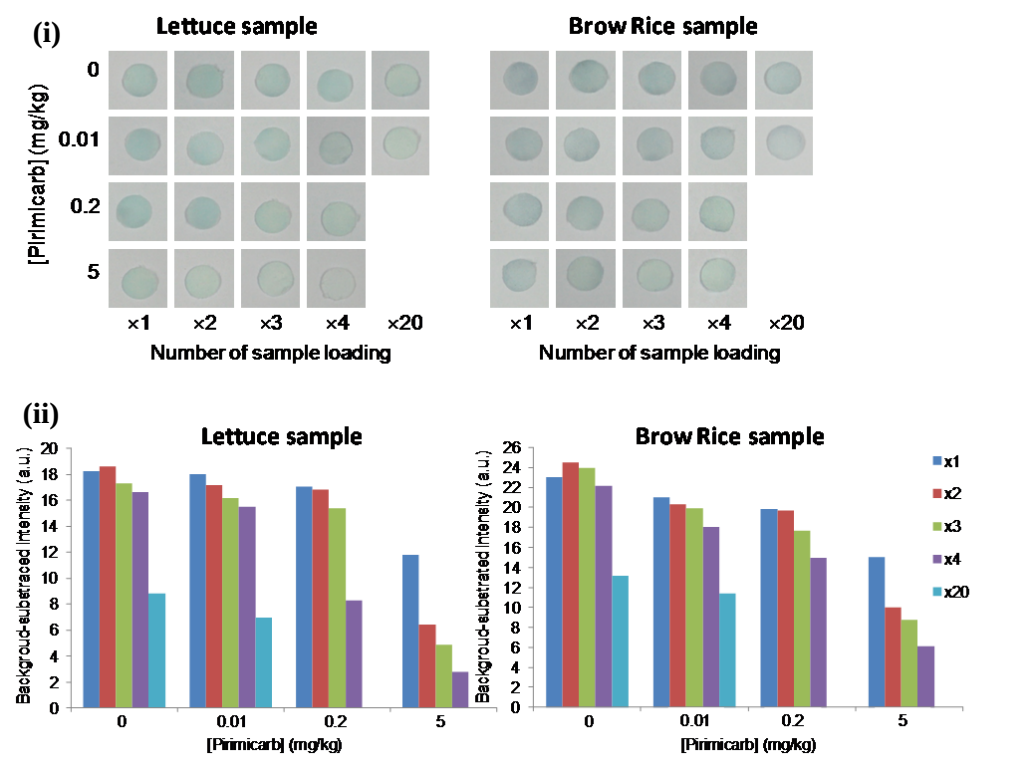
(i) Photographs and (ii) plots of the mean intensity of the pre-concentration method with multiple applications of 20 μL of 0.01, 0.2, and 5 mg/kg spiked pirimicarb in lettuce and brown rice

The paper-based sensor is able to absorb a high amount of solution in the sensing area. For this reason, the analyte can be instantaneously concentrated on the paper by repeatedly loading the sample solution on the test zone. The performance of the self-contained pre-concentration method was investigated in the detection of pirimicarb in spiked lettuce and brown rice samples at 0, 0.01, 0.2 and 5 mg/kg concentrations. Then, 10 µl of sample solution was repeatedly applied to the test zone for 1, 2, 3, 4 and 20 times at 5-min intervals. The results are shown in Figure 8(Fig. 8). The increasing number of sample application resulted in the decreasing color intensity of the test zone. The color intensity change compared with the negative control could be clearly distinguished by the naked eye with 4 applications of 0.2 and 5 mg/mL of pirimicarb in spiked lettuce and brown rice samples, respectively. The self-contained pre-concentration method provided good detection sensitivity toward pirimicarb (concentrations as low as 0.2 mg/kg), which was sufficiently sensitive for the screening of pirimicarb residues at MRLs of 5 mg/kg in lettuce for EU standards and 0.3 and 1 mg/kg in brown rice and lettuce, respectively, for JA standards. The total analysis time was 45–60 min, which was adequate for home usage. For sample concentrations as low as a default MRL of 0.01 mg/kg, a sufficient signal could be obtained by the repetitive application of the sample up to 20 times. Nevertheless, the analysis took approximately 3 hours due to the slow absorption of the sample solution on the paper.

To cope with such a limitation, the sequential heating of the paper-based sensor after each cycle of sample application was proposed to accelerate the dehydration and absorption processes. The effect of temperature on the enzymatic reaction on the paper-based sensor was investigated. The color intensity of the hydrolyzed indoxyl acetate decreased when the paper-based sensor was heated above 45 °C on the hotplate. This indicated that the AChE activity and the substrate stability were influenced by temperature. The heating method was then performed at 40 °C for the determination of 0.01 mg/kg pirimicarb residue in lettuce and rice samples. The color change was visibly distinguishable when the total analysis time was reduced to approximately 40 min. Thus, the pre-concentration method was successfully improved to enhance the limit of detection, and heating approaches can be included to reduce the analysis time.

These findings demonstrated the potential application of the paper-based AChE inhibition assay combining a wet assay developed herein for the screening of OP and CM residues in real samples. To enhance the performance of the developed device for the detection of OP and CM residues at low concentrations below 5 mg/kg, the effective extraction process or the pre-concentration method were required.

### Storage stability

To determine the storage stability, the developed device spotted with ACE and indoxyl acetate was stored at room temperature (~27 °C), 4 °C, and –20 °C for multiple days. The enzyme and substrate immobilized on paper were stable, and retained almost 85 % of efficient activity for up to three months, one week and two days of storage at -20 °C, 4 °C and room temperature, respectively. The stability of the substrate is an important consideration for extending shelf life. The storage stability can be enhanced by keeping the device in aluminum foil zipped under inert atmosphere and stored in the freezer to prevent exposure from light, air and moisture. 

## Conclusion

The paper-based sensor with wet assay developed herein provides a simple and rapid screening method for pesticide detection. Measuring the samples requires only an addition of the sample followed by the application of a buffer solution after an incubation step. The pesticide residues can be observed by the naked eye or with an image processing program as a result of the color change produced by the reaction between AChE and indoxyl acetate. The limitation of detection for OPs and CMs ranged between 0.005 and 0.1 ppm, depending on the pesticide. The assay was successfully applied in the screening of OP and CM residues in agricultural products with a LOD of 5 mg/ml by the naked eye. The sample preparation by solvent extraction and/or the pre-concentration method can be carried out to enhance the sensitivity of the device in detecting pesticide residues at a default MRL of 0.01 mg/kg. The paper-based sensor combined with the wet assay provides an alternative tool for the convenient screening of pesticide residues in food and environment with low cost, minimized reagent consumption, and simple operation by non-trained personnel. The protocols herein also aimed to enhance the sensitivity of paper based-devices for the measurement of other assays.

## Notes

Tanawut Tantimongcolwat and Virapong Prachayasittikul (E-mail: virapong.pra@mahidol.ac.th; Tel.: +66 2 419 7172, Fax: +66 2 441 4380) contributed both as corresponding authors.

## Acknowledgements

The authors gratefully acknowledge Prof. Dr. Sompon Wanwimolrukt and Ms. Onnicha Kanchanamayoon at the Center for Innovation Development and Technology Transfer, Faculty of Medical Technology, Mahidol University for their supports in GC-MS/MS measurement and Prof. Orawon Chailapakul, Department of Chemistry, Faculty of Science, Chulalonkorn University for her great assistance. This project is supported by Mahidol University. A.A. gratefully acknowledges support from Young Scholars Research Fellowship from The Thailand Research Fund (grant no. TRG5780015). 

## Figures and Tables

**Table 1:**
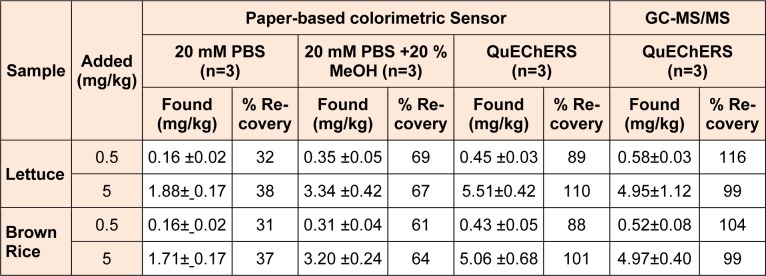
Determination of the spiked pirimicarb at 0.5 and 5 mg/kg in rice and lettuce samples with different sample preparation procedures using 20 mM PBS pH 7.4, 20 mM PBS pH 7.4 + 20 % methanol, and QuEChERS-extraction by the developed method and compared with GC-MS/MS
